# Research progress on the role of microbiota in the pathogenesis of gallstone disease

**DOI:** 10.3389/fmicb.2025.1672767

**Published:** 2025-10-08

**Authors:** Yuntian Liu, Bihui Yao, Xiaoqin Yang, Qi Sun, Xusheng Yang, Lu Liang

**Affiliations:** ^1^Affiliated Baotou Clinical College of Inner Mongolia Medical University, Baotou, China; ^2^Hepatobiliary Surgery Department, Baotou Central Hospital, Baotou, China

**Keywords:** gallstone disease, biliary microbiota, gut microbiota, β-glucuronidase, biofilm, bile acid dysmetabolism

## Abstract

Gallstone disease (GSD) is a prevalent digestive disorder traditionally believed to stem from disturbances in cholesterol metabolism and imbalances in bile composition. Recent evidence highlights a shift in understanding GSD from a primarily metabolic disorder to a microbial-mediated pathology. The biliary tract, rather than a sterile environment, may harbor a distinct microbial community that, under homeostatic conditions, may coexist with the host to maintain biliary health. Disruption of this equilibrium can initiate GSD. Gut microbiota contributes to GSD by modulating enterohepatic circulation via the FXR-FGF15 pathway and producing metabolites, including β-glucuronidase, that promote cholesterol precipitation. Biofilm formation by biliary microbes further enhances nucleation and gallstone formation. Recent studies have characterized biliary microbial communities but are limited by small sample sizes, methodological heterogeneity and scant mechanistic insight. These limitations impede translation into clinical practice. Despite these limitations, accumulating evidence underscores the potential of targeting biliary and intestinal microbiota in GSD prevention and therapy. This review integrates current evidence to elucidate microbiota-mediated mechanisms and translational opportunities, offering an innovative perspective for preventive and therapeutic strategies.

## Introduction

Gallstone disease (GSD), also known as cholelithiasis, refers to the formation of stones within the biliary system, including the gallbladder (the major phenotype) as well as intra- and extrahepatic bile ducts. This frequently-occurring clinical condition is among the most prevalent disorders encountered in hepatobiliary surgery (Marschall and Einarsson, 2007; Unalp-Arida and Ruhl, 2024). Fundamentally, GSD may stem from the imbalanced proportions of organic solutes (e.g., bilirubin, bile salts, phospholipids, and cholesterol) in bile, leading to the precipitation of solid components (Reshetnyak, 2012; Sun et al., 2022). According to the specific composition, GSD is classified into cholesterol gallstones, and pigment stones (black and brown) (Kumar et al., 2021). The estimated prevalence of GSD in the general population reaches approximately 10–15% (Lysandra et al., 2022). The prevalence of GSD continues to rise due to rapid economic development, dietary changes, and population aging, making it an important public health concern. GSD can lead to various complications, such as biliary colic, cholecystitis, cholangitis, and pancreatitis (Costanzo et al., 2023), significantly compromising patient quality of life and increasing healthcare burdens.

Microorganisms are ubiquitous in the human body, with bacteria being the most abundant and forming stable symbiotic communities, particularly in the gastrointestinal (GI) tract (Hou et al., 2022). Bile was traditionally considered sterile due to conventional culture results and the presumed antimicrobial properties of bile and the sphincter of Oddi (Begley et al., 2005; Csendes et al., 1996). However, organisms such as *Escherichia coli* (*E. coli*), *Klebsiella pneumoniae*, and *Enterococcus* spp. have been detected subsequently in bile and gallstone specimens, through culture-based methods and polymerase chain reaction (PCR) techniques, indicating that the biliary tract may harbor a resident or transient microbiota (Pratt and Kolter, 1998; Swidsinski et al., 1998; Wang et al., 2023). However, culture-dependent methods exhibit limited sensitivity for bacterial identification, which may be explained primarily by the coexistence of diverse microbial species in the biliary tract that may constrain the comprehensive characterization of the biliary microbiota. With the advancement of research and the widespread adoption of high-throughput sequencing technologies in human microbiome studies, our understanding of the human microbiota has been enhanced significantly owing to non-culture-based molecular approaches such as 16S rRNA gene sequencing and metagenomics (Wensel et al., 2022; Zhang et al., 2024). In particular, these methods have enabled the identification of previously unrecognized biliary microorganisms when applied to biliary research, drawing increasing attention to the role of biliary microbiota in GSD and propelling research on GSD pathogenesis into a new microbially oriented paradigm (Hu et al., 2024; Tan et al., 2025).

## Pathogenesis of GSD

Gallstone disease has traditionally been explained by a tripartite model: supersaturation of bile with cholesterol, impaired gallbladder motility, and enhanced nucleation (Carey, 1993; Di Ciaula et al., 2018). Supersaturation occurs when the balance among cholesterol, bile acids, and phospholipids is disrupted, leading to cholesterol crystal precipitation (Di Ciaula et al., 2018). Impaired gallbladder motility delays bile emptying, prolonging crystal retention and facilitating aggregation (Carey, 1993). Meanwhile, mucin hypersecretion provides a pronucleating matrix that accelerates crystal growth and stone formation (Di Ciaula et al., 2018). These mechanisms also underlie the classification of gallstones into cholesterol and pigment types, with the latter often associated with hemolysis, cirrhosis, or biliary infection (Di Ciaula et al., 2018).

Emerging evidence indicates that microbial factors, particularly dysbiosis of the gut and biliary microbiota, may interact with these classical lithogenic pathways (Banerjee et al., 2024; Hu et al., 2022; Hu et al., 2024; Tan et al., 2025). Microbial alterations may influence bile composition, cholesterol solubility, and mucin expression, thereby facilitating crystal nucleation and stone formation (Hu et al., 2022; Hu et al., 2024; Tan et al., 2025). While the precise mechanisms are complex and under active investigation, these findings highlight the importance of considering microbial contributions to GSD, setting the stage for a focused discussion of biliary microbiota dysbiosis.

## Relationship between biliary microbiota dysbiosis and GSD

Recently, a distinct microbial community, termed the biliary microbiota, has been identified to reside in the biliary tract. Under physiological conditions, the biliary microbiota maintain a dynamic homeostasis with the host. Nevertheless, disruption of this balance, known as dysbiosis, is implicated in the development of biliary diseases and GSD. Zhang et al. (2024). Historically, obtaining viable biliary samples has been challenging, resulting in scarce and less comprehensive research compared with studies of the gut or skin microbiota, which has limited our understanding of the normal biliary microbial community. Recent large-scale clinical cultivation and detection analyses, however, have provided a more robust data foundation for investigating the biliary microbiota (Zheng et al., 2024).

In 2014, Jiménez et al. (2014) performed the first microbiota analysis of bile, gallbladder mucus, and gallbladder tissue biopsies from healthy pigs, identifying *Proteobacteria*, *Firmicutes*, and *Bacteroidetes* as the dominant bacterial phyla. These results suggested that the gallbladder harbors its own specific microbiota and raised the critical question of whether the biliary microbial communities originate from resident bacteria or from other anatomical sites.

This question has generated an ongoing debate regarding the origin of the biliary microbiota. One school of thought supports the residency hypothesis, proposing that the biliary tract harbors a relatively stable, self-sustained microbial community (Nicoletti et al., 2020). In contrast, the reflux origin hypothesis suggests that microbes detected in bile largely derive from retrograde translocation from the duodenum or oral cavity (Han et al., 2021). Current evidence is inconclusive: sequencing studies identifying consistent taxa across individuals favor the residency hypothesis, whereas clinical observations and experimental models showing overlap with duodenal microbiota support the reflux hypothesis. These conflicting findings underscore methodological heterogeneity and highlight the need for longitudinal studies with carefully selected control samples to distinguish true residents from transient colonizers.

Given the ethical and practical limitations of sampling bile from healthy individuals, many studies have adopted alternative approaches. Specifically, bile samples from patients with non-inflammatory gallbladder polyps or from liver transplant donors without hepatobiliary disease are commonly utilized as surrogate controls, providing the closest possible representation of normal biliary microbiota and composition. Higashi et al. (2022) demonstrated the reflux of bile from the bile ducts into the gallbladder. With the use of cine-dynamic magnetic resonance cholangiopancreatography with a spatially selective inversion-recovery pulse, Ito et al. (2014) reported that retrograde bile movement occurred as a physiologic phenomenon in healthy controls, which was found in 74% (26 out of 35) of the nondilated group. Accordingly, we hypothesize that the biliary microbiota encompasses microbial communities residing in the gallbladder as well as in the intrahepatic and extrahepatic bile ducts. Phylum-level analysis using 16S rRNA gene sequencing or metagenomic provides a broad overview of microbial composition and trends. In contrast, genus-level composition and diversity typically indicate the specific microenvironmental conditions of individual anatomical niches (Gwak and Rho, 2020). Therefore, our research continued to carry out statistical analyses of bacterial community composition at both the phylum and genus levels across all included studies.

Molinero et al. (2019) analyzed gallbladder bile samples from 13 liver transplant donors without hepatobiliary disease and 14 patients with GSD using 16S rRNA gene sequencing. They found that the dominant phyla in healthy donors were *Actinobacteria*, *Firmicutes*, *Bacteroidetes*, and *Proteobacteria*, while major genera included *Sphingomonas* and *Methylobacterium* (Molinero et al., 2019). This study provided baseline evidence that the biliary tract may harbor a resident microbiota in healthy controls, laying the groundwork for the identification of GSD-associated dysbiosis patterns (Molinero et al., 2019). Xiao et al. (2024) pioneered the integration of 16S rRNA gene sequencing and LC–MS–based metabolomics in comparing patients with asymptomatic gallbladder polyps and those with common bile duct (CBD) stones. Consequently, polyp patients exhibited a microbiota composition similar to healthy controls, while stone patients showed decreased *Actinobacteria*, increased *Proteobacteria*, and elevated *Enterococcus* abundance, implying a possible synergy between microbial and metabolic alterations in GSD pathogenesis (Xiao et al., 2024). Similarly, Wu et al. (2013) reported comparable findings in 29 Chinese patients with GSD, identifying six core bacterial phyla—primarily *Actinobacteria*, *Bacteroidetes*, *Firmicutes*, and *Proteobacteria*—and noting *Bacteroides* as the most abundant genus. This study offered important population-based evidence and facilitated the clarification of conserved microbial signatures across individuals with GSD (Wu et al., 2013). In another study on biliary obstruction, Kim et al. (2021) documented significantly increased *Enterococcus* levels in the bile of patients with brown pigment stones compared to those with non-stone obstructions. Moreover, patients with GSD may also exhibit significantly reduced biliary microbial diversity compared to individuals without hepatobiliary disorders. Xiao et al. (2021) reported significantly reduced microbiota diversity in the bile of patients with intrahepatic bile duct stones. Similarly, Molinero et al. (2019) observed markedly lower microbiota diversity in patients with GSD compared to liver transplant donors without hepatobiliary disease. Consistently, Xiao et al. (2024) also observed significantly reduced microbial richness, diversity, and evenness in subjects with bile duct stones compared to the control (patients with gallbladder polyps).

Tables 1, 2 summarize the results of recent investigations into the human biliary microbiota, allowing us to derive the following principal insights:

(1)    GSD patients and healthy controls exhibit markedly different taxonomic composition of the biliary microbiota at both the phylum and genus levels. At the phylum level, both healthy and GSD-affected biliary microbiota are dominated by *Firmicutes*, *Proteobacteria*, *Bacteroidetes*, and *Actinobacteria*. However, there are significant differences in their relative abundances. To be specific, the biliary microbiota of GSD patients exhibits a higher proportion of *Firmicutes*, *Bacteroidetes*, and *Proteobacteria*, while *Actinobacteria* and other phyla are comparatively underrepresented. At the genus level, dominant taxa in the normal biliary tract include *Ruminococcus, Akkermansia*, *Sphingomonas*, *Pseudomonas*, and *Escherichia*. As proposed by Lyu et al. (2021), *Pseudomonas* and *Escherichia* may be part of the normal bile microbiota. In contrast, the dominant genera in GSD patients differ significantly from those in healthy controls, with higher abundances of *Escherichia*, *Enterococcus*, *Pseudomonas*, *Enterobacter*, *Klebsiella*, *Streptococcus*, and *Bacteroides*.
(2)    GSD is also characterized by markedly altered microbial diversity, in addition to compositional shifts. For instance, microbiota of GSD patients displays significantly reduced diversity, as compared to the normal biliary microbiota (as observed in patients with gallbladder polyps or liver transplant donors without hepatobiliary disease). The enrichment of single or few bacterial taxa may be associated with bile metabolic disturbances, bile stasis, or inflammatory responses. Indeed, patients with GSD are frequently observed with reduced microbial diversity, further longitudinal studies are needed to elucidate whether this alteration serves as a causal factor or a consequence of GSD.

**TABLE 1:** Summary of studies on the relative normal bile duct microbiota.

Literature	Sample source	Research technique	Research sample	Sampling method	Dominant microbiota of the biliary tract
Xiao et al., 2024	Patients with gallbladder polyps	16S rRNA + LC-MS	Gallbladder bile	Surgery	Dominant phyla: *Firmicutes*, *Proteobacteria*, *Bacteroidetes*, and *Actinobacteria* Dominant genera: *Ruminococcus*, *Mucus-forming bacillus*, and *Muribaculaceae*
Molinero et al., 2019	Liver transplant donors	16S rRNA + WMS	Gallbladder bile	Surgery	Dominant phyla: *Actinobacteria*, *Firmicutes*, *Bacteroidetes*, and *Proteobacteria* Dominant genera: *Sphingomonas*, and *Methylobacterium*
Lyu et al., 2021	Patients without liver or biliary diseases	16S rRNA	Biliary bile	Endoscopic retrograde cholangiopancreatography (ERCP)	Dominant phyla: Proteobacteria, Actinomycetes, and Bacteroidetes Dominant genera: *Pseudomonas, E. coli*-*Shigella*

**TABLE 2:** Summary of studies on the microecology of the biliary tract in patients with GSD.

Literature	Sample source	Research technique	Research sample	Sampling method	Dominant microbiota of the biliary tract
Xiao et al., 2024	Patients with GSD	16S rRNA + LC-MS	Bile ducts	ERCP	Common phyla: *Firmicutes*, *Ascomycetes*, and *Actinomycetes* Common genera: *Ruminococcus, Mucor, Enterococcus*, and *Anaplasma*
Wu et al., 2013	Patients with GSD	16S rRNA	Gallbladder bile	Surgery	Dominant phyla: *Firmicutes*, *Actinomycetes*, *Ascomycetes*, and TM7 Dominant genera: *Enterobacteriaceae, Pseudomonas, Propionibacteria*, and *Clostridia*
Kim et al., 2021	Patients with bile duct stones	16S rRNA	Bile ducts	ERCP	Dominant phyla: Ascomycetes, *Firmicutes*, *Anaplasma, Clostridia*, and *Actinomycetes* Dominant genera: *Enterobacteriaceae, Pseudomonas, E. coli - Shigella*, and *Enterococci*
Xiao et al., 2021	Patients with intrahepatic bile duct stones	16S rRNA	Bile ducts	Hepatectomy	Dominant phyla: *Ascomycetes*, and *Actinomycetes* Dominant genera: *Enterococci, Enterobacteriaceae*, and *P. aeruginosa*
Molinero et al., 2019	Patients with GSD	16S rRNA + whole-metagenome sequencing (WMS)	Gallbladder bile	Surgery	Dominant phyla: Actinobacteria, *Firmicutes*, *Bacteroidetes*, and *Ascomycetes* Dominant genera: *Anaplasma, E. coli - Shigella*
Lyu et al., 2021	Patients with bile duct stones	16Sr RNA	Bile ducts	ERCP	Dominant phyla: *Ascomycetes*, *Firmicutes*, and *Actinomycetes* Dominant genera: *Pseudomonas, E. coli - Shigella*
Xie et al., 2022	Patients with GSD	16Sr RNA	Gallbladder bile	Surgery	Dominant phyla: Ascomycetes, *Firmicutes*, and *Actinomycetes* Dominant genera: *Fusobacterium, Anaplasma*, and *Mycobacterium spp.*
Saab et al., 2021	Patients with bile duct stones	16S rRNA	Bile ducts	ERCP	Dominant phyla: *Ascomycetes*, *Firmicutes*, *Actinomycetes*, and *Mycobacteriophages* Dominant genera: *Enterococcus, Streptococcus*, and *Lactobacillus*
Ye et al., 2016	Patients with bile duct stones	16S rRNA	Bile ducts	ERCP	Dominant phyla: *Ascomycetes*, *Firmicutes*, *Actinomycetes* Dominant genera: *Streptococcus, Anaplasma*, *Veronococcus*, and *Prevotella, Rothschilds E. coli, Klebsiella*, and *unclassified Enterobacteriaceae genera*
Shen et al., 2015	Patients with bile duct stones	16S rRNA + WMS	Bile ducts	ERCP	Dominant phyla: *Ascomycetes*, *Firmicutes*, and *Actinomycetes* Dominant genera: *E. coli, Klebsiella, Anaplasma*, *Enterococcus*, and *Pseudomonas*
Han et al., 2021	Patients with bile duct stones	16S rRNA	Bile ducts	Surgery	Dominant phyla: *Ascomycetes*, *Firmicutes*, and *Actinomycetes* Dominant genera: *E. coli - Shigella, Clostridium*, and *Enterococci*

Altogether, these findings raise an important question: how do specific biliary bacteria functionally contribute to GSD formation, beyond compositional shifts? An in-depth explanation of this issue will contribute to bridging the gap between correlation and causation.

## Roles and mechanisms of specific biliary bacteria in the formation of GSD

Gallstone disease can be classified into cholesterol gallstones and pigment stones, with the latter further divided into black pigment stones and brown pigment stones (Kumar et al., 2021). The formation of cholesterol gallstones is primarily attributed to bile composition imbalances—particularly cholesterol supersaturation—gallbladder dysmotility, and accelerated nucleation (Wang K. et al., 2025). In contrast, pigment stones are generally associated with hemolytic anemia, liver cirrhosis, and biliary tract infections (Lysandra et al., 2022). Traditionally, cholesterol gallstones were regarded as a consequence of metabolic disturbances. However, emerging evidence suggests that alterations in the biliary microbiota may also contribute to their development (Binda et al., 2022). Historically, the role of biliary bacteria has received little attention in GSD formation. Nevertheless, a potentially active role for microorganisms in GSD pathogenesis has been underscored by the frequent detection of bacteria in the biliary tract, with the rapid advancement of high-throughput sequencing and metagenomic technologies. Notably, specific bacterial taxa and their secreted products not only gain a colonization advantage within the biliary environment but also modulate bile composition, promote crystal nucleation, facilitate biofilm formation, and trigger local inflammation (Liu et al., 2025). Together, these mechanisms collectively drive gallstone formation. Our subsequent discussion will focus on the roles and mechanisms of major biliary bacterial phyla and representative genera in the pathogenesis of GSD. To provide a concise synthesis, we summarized the main bacteria, their key products, mechanisms, and related stone types in Table 3. This table complements the detailed descriptions in the text and improves readability.

**TABLE 3:** Bacteria, key products, mechanisms, and related gallstone types.

Bacteria	Key products	Main mechanisms	Stone type(s)
*E. coli*	β-G, PLs, BSH, biofilm	Bilirubin hydrolysis, cholesterol supersaturation, biofilm stabilization	Pigment, cholesterol
*H. pylori*	Urease, CagA, ROS	Oxidative stress, mucosal damage, nidus formation	Cholesterol
*P. aeruginosa*	β-G, PLA2, biofilm	Strong biofilm, bile acid alteration, nucleation	Cholesterol, pigment
*Enterococcus*	BSH, β-G, biofilm	Bile acid dysregulation, mucin secretion, colonization	Cholesterol, pigment
*C. perfringens*	β-G, PLC	High β-G activity, bile component hydrolysis	Pigment
*Bacteroides*	β-G, BSH, LPS	Cholesterol supersaturation, inflammation, biofilm	Cholesterol, pigment
*Actinomyces*	SCFAs	Protective role; depletion weakens barrier	Cholesterol
*Desulfovibrionales*	H2S, secondary BAs	Increase bile hydrophobicity, increase cholesterol secretion	Cholesterol

### Proteobacteria

**FIGURE 1:**
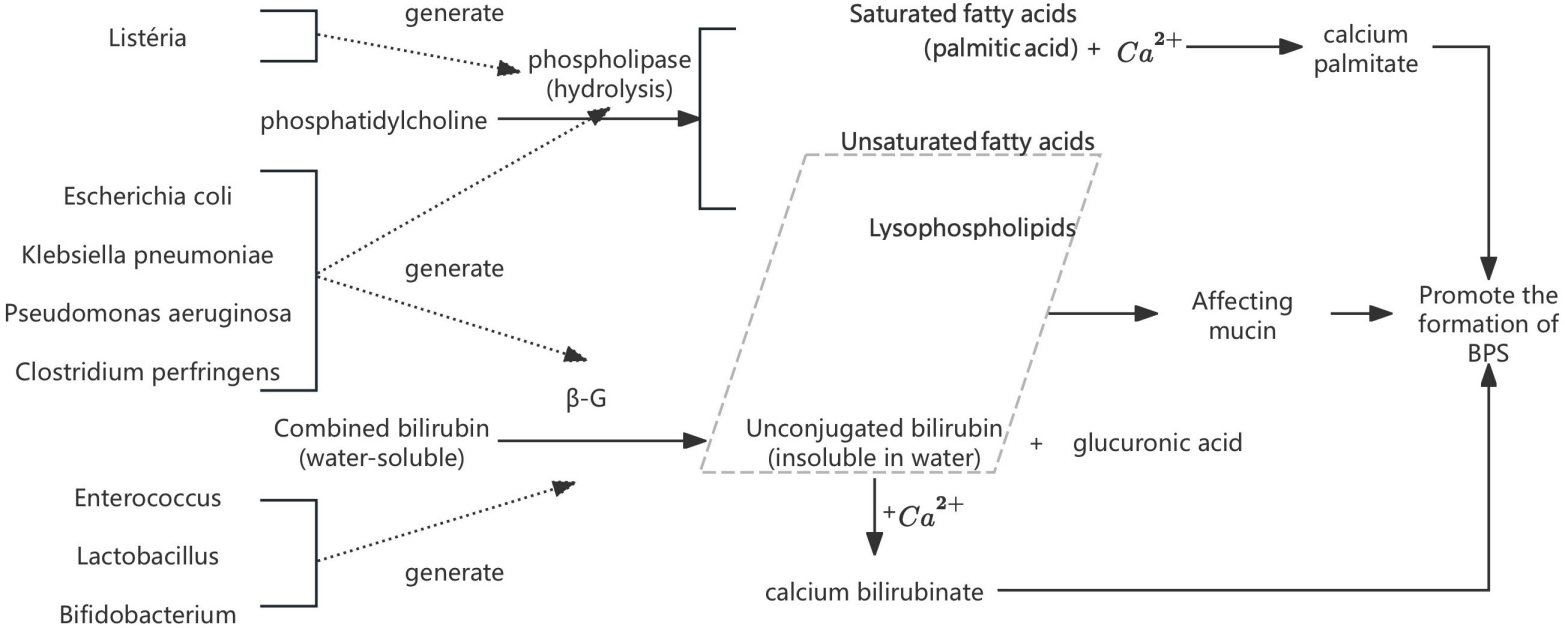
The mechanism by which bacteria such as *E. coli* promote stone formation.

***Escherichia coli* (*E. coli*).** Bacterial infection has long been recognized as a key contributor to pigment gallstone pathogenesis. As early as 1966, Japanese researcher Maki (Maki, 1966) proposed the seminal hypothesis that *E. coli* participates in calcium bilirubinate stones through the production of β-glucuronidase (β-G) and phospholipases (PLs). Subsequent studies confirmed that β-G hydrolyze conjugated bilirubin (CB) into unconjugated bilirubin (UCB), which readily binds to Ca^2+^ to form insoluble calcium bilirubinate (Nakano et al., 1988). Meanwhile, bacterial PLs degrade phosphatidylcholine (PC) into palmitic acid and lysolecithin: the former precipitates with Ca^2+^ to form calcium palmitate, while the latter interacts with mucin and bilirubin to promote calcium phosphate crystallization (Hattori et al., 2000). Both processes act synergistically to drive brown pigment stones development (Xiao et al., 2021; Figure 1). These findings collectively highlight the mechanistic role of bacterial enzymes in modulating bile composition and initiating stone nucleation, which has been validated *in vitro* by Higashijima et al. (1996) and Leung et al. (2001). Xiao et al. (2021) further proposed that other common biliary bacteria [e.g., *Enterococcus, Clostridium perfringens* (*C. perfringens*), and *Pseudomonas aeruginosa* (*P. aeruginosa*)] can also produce β-G, thereby contributing to GSD. These species will be listed in subsequent sections without further elaboration. Recent studies have corroborated these findings, identifying E. coli and other biliary bacteria as significant contributors to gallstone formation through their enzymatic activities and biofilm formation capabilities (Muturi et al., 2024; Tan et al., 2025; Zhang et al., 2025).

Recent evidence suggests that *E. coli* also contributes to cholesterol gallstone pathogenesis (Li et al., 2022). Cholesterol is a bile-excreted metabolic product of lipid metabolism *in vivo*. As a nonpolar molecule, cholesterol is poorly soluble in water, even though water constitutes approximately 85% of the bile (Tateyama and Matsushiro, 1981). Bile salts and phospholipids form bile salt–phospholipid–cholesterol complexes, also known as micelles, to maintain cholesterol solubility, eventually facilitating cholesterol solubilization (Yeo et al., 2023). Water-insoluble cholesterol crystals may be formed when these three key components are disrupted or distributed unevenly (Chatterjee and Irani, 2024). As discussed above, *E. coli* can survive and proliferate in infected bile. The phospholipase it produces can hydrolyze a substantial portion of bile phospholipids, which may increase the relative concentration of cholesterol, leading to cholesterol supersaturation and subsequent crystal precipitation, two key steps in triggering cholesterol gallstone development (Liu et al., 2025). Moreover, *E. coli* can also secrete bile salt hydrolases (BSH) and 7α-dehydroxylases, resulting in weakened capacity of emulsifying bile salts and boosting cholesterol precipitation (Berr et al., 1996). Furthermore, with the additional ability to form biofilms in the biliary system, it can also stabilize nascent cholesterol crystals and enhance nucleation (Swidsinski et al., 2005). Collectively, these findings support a multifactorial microbial contribution to lithogenesis, challenging the traditional notion that bacteria are irrelevant in cholesterol gallstone formation.

In addition to its enzymatic activity, *E. coli* can employ the AcrAB-TolC efflux pump [i.e., a resistance-nodulation-division (RND) family transporter] to expel toxic bile salts and fatty acids, thereby enhancing bacterial survival in the biliary system (Gaurav et al., 2023; Jang, 2023). Okusu et al. (1996) demonstrated that this efflux pump could also facilitate sustained colonization, in addition to strengthening bile salt tolerance. This persistent colonization, in turn, can exert functional roles in the formation of GSD. In addition to these effects, the AcrAB-TolC efflux pump is also involved in biofilm formation, enhanced virulence during infection, and bacterial adaptation to environmental stressors (Langevin and Dunlop, 2018; Pérez et al., 2012; Yamasaki et al., 2015), all of which may indirectly contribute to the pathogenesis of GSD. Therefore, colonization represents both a survival strategy and a mechanistic contributor to the formation of GSD. Recent studies have highlighted the significance of the AcrAB-TolC efflux pump in bacterial adaptation to the biliary environment and its potential as a therapeutic target (Jang, 2023; Tan et al., 2025).

***Helicobacter pylori* (*H. pylori*).** *H. pylori* is a common bacterium that can live in the GI tract, which has been demonstrated to exhibit a potential association with GSD. Two prevailing hypotheses suggest that *H. pylori* may reach the biliary system via either duodeno-biliary reflux or the portal venous circulation (Helaly et al., 2014; Waluga et al., 2015). It is established that *H. pylori* can be present in the biliary tract, regardless of the pathway. Azimirad et al. (2023) identified *H. pylori* in bile via 16S rDNA sequencing. In another study examining gallbladder tissues from 94 symptomatic GSD patients, Attaallah et al. (2013) detected *H. pylori* in 35 patients (37%) by at least one method of urease testing, Giemsa staining, and immunohistochemistry. A large multi-center retrospective study by Yao et al. (2024), involving over 70,000 healthy controls, concluded a positive correlation between *H. pylori* infection and GSD. These findings were further supported by Wang et al. in a meta-analysis (Wang et al., 2021) that the prevalence of *H. pylori* infection in the gallbladder was significantly higher in patients with chronic cholecystitis and GSD compared to controls without biliary diseases (23.7% vs. 7.23%, *P* < 0.0001), suggesting a strong positive association between *H. pylori* colonization and the risk of GSD. Moreover, studies by Zhang et al. (2015) and Takahashi et al. (2014), involving 15,523 and 15,551 subjects respectively, assessed GSD prevalence following *H. pylori* eradication. Both studies reported significantly lower GSD prevalence in individuals following *H. pylori* eradication compared to those who remained *H. pylori*-positive (9.02% vs. 9.47%, *p* < 0.0001 (Zhang et al., 2015); 6.08% vs. 4.73%, *p* < 0.0001 (Takahashi et al., 2014), further supporting a potential causal relationship. A review of existing studies suggests that we can conclude several mechanisms by which *H. pylori* in the biliary tract may contribute to GSD pathogenesis as follows:

(1) *H. pylori* infection in the gallbladder can induce oxidative stress via reactive oxygen species (ROS) and reactive nitrogen species (RNS), along with the release of proinflammatory cytokines (e.g., IL-1, IL-6, and TNF-α), all of which are implicated in GSD pathogenesis (Binda et al., 2022; Sipos et al., 2001). Prior investigation has documented that oxidative stress can alter bile composition by promoting the biosynthesis of hepatic cholesterol, increasing hydrophobic bile acids, and enhancing phospholipase activity, leading to bile supersaturation and increased viscosity synergistically (Kasprzak et al., 2015). Administration of IL-1 and TNF-α in mice has been shown to elevate serum cholesterol levels and upregulate HMG-CoA reductase expression at both transcriptional and translational levels, thereby promoting cholesterol biosynthesis (Maurer et al., 2009). Exposure of cultured human gallbladder epithelial cells to TNF-α and IL-1 also increases susceptibility to GSD formation by disrupting sodium and chloride transport, and impairing absorptive function (Rege, 2000). CagA, a major virulence factor of *H. pylori*, can induce the formation of neutrophil extracellular traps (NETs) through ROS- and peptidylarginine deiminase 4 (PAD4)-dependent pathways, facilitating the release of chromatin networks. These NETs provide nucleation platforms for cholesterol crystal deposition, and, via the release of myeloperoxidase and other proinflammatory mediators, exacerbate biliary inflammation, increase bile viscosity, and promote cholesterol supersaturation, eventually triggering the formation of GSD (Muñoz et al., 2019). In addition, CagA can directly cause damage to the mechanical barrier of the gallbladder epithelium. Yu et al. (2024a) demonstrated that intra-gallbladder delivery of CagA in mice led to mucosal disruption, epithelial detachment, and necrosis. Further mechanistic insights suggest that CagA disrupted tight junctions and epithelial polarity, inducing inflammation and apoptosis (Yu et al., 2024a). This epithelial damage increases mucosal permeability, impair bile reabsorption, and promote bile stasis, thereby creating a stone-promoting microenvironment favorable to GSD development (Yu et al., 2024a).

(2) *H. pylori* may promote the formation of GSD through several additional mechanisms, beyond inducing oxidative stress and inflammatory responses. First, urease-positive *H. pylori* strains have been shown to hydrolyze urea into ammonia and bicarbonate, thereby increasing local pH and promoting the precipitation of insoluble calcium salts by interacting with carbonate and other anions (Belzer et al., 2006). These salts may serve as nucleation sites for facilitating the deposition of cholesterol and bilirubin (Belzer et al., 2006). However, this effect appears to present with individual variations, possibly due to differences in bile buffering capacity, bacterial load, or strain-specific urease activity. Moreover, some researchers hypothesized *H. pylori* itself might act as a nidus for lithogenesis, functioning as a foreign body around which bile components aggregate to initiate lithogenesis (Grigor’eva and Romanova, 2020). In addition, *H. pylori* infection has been found to be associated with increased endogenous β-G activity, further facilitating bilirubin precipitation and pigment stone formation—a mechanism detailed in the previous text (Mo, 2016). Together, *H. pylori* exerts multifaceted roles in GSD pathogenesis, beyond inflammation and oxidative injury.

***P. aeruginosa.*** *P. aeruginosa* is a dominant species in the biliary tract of patients with GSD. In a 2024 multicenter study comparing bile microbiota in choledocholithiasis and gallbladder polyp patients, *P. aeruginosa* biofilm was observed to be significantly more metabolically active in the choledocholithiasis group and closely associated with stone formation (Xiao et al., 2024). *P. aeruginosa* can modulate the size and composition of the bile acid pool through multiple pathways, creating a favorable environment for GSD formation (Wang et al., 2024). Lyu et al. (2021) analyzed the biliary microbiota of 15 patients with primary CBD stones and 4 individuals without biliary disease using 16S rRNA gene sequencing. Their findings further revealed that *P. aeruginosa* was a dominant species in the bile of GSD patients, in contrast to its absence or very low abundance in healthy controls (Lyu et al., 2021). Peng et al. (2015) investigated the taxonomic composition and functional characteristics of bacteria present in cholesterol gallstones and bile. They identified 30 bacterial genera in the GSD and only 2 in the bile, among which *P. aeruginosa* was confirmed to be the predominant genus associated with cholesterol gallstones (Peng et al., 2015). Among all strains detected in the biliary tract, *P. aeruginosa* exhibited the highest β-G activity and the highest phospholipase A2 (PLA2) expression (Grigor’eva and Romanova, 2020).

Typically, microorganisms aggregate at interfaces to form multispecies communities, rather than existing in isolation. Biofilms are a group or cluster (i.e., aggregates) of bacteria embedded in extracellular polymeric substances (EPS), which can form within the biliary tract (Montanari et al., 2025). EPS are composed mainly of polysaccharides, proteins, nucleic acids, and lipids, serving as the structural scaffold of the biofilm, which is pivotal for adhesion, protection, and metabolic activity (Flemming and Wingender, 2010). During the formation of GSD, bacteria can secrete EPS to establish biofilms that facilitate stable colonization of the biliary epithelium or bile. Under pathological conditions, these biofilms may transition from a benign to a lithogenic phenotype. By binding with Ca^2+^ and UCB, EPS can create a nucleation platform for compounds such as calcium bilirubinate, thereby promoting crystal aggregation and GSD initiation. Moreover, it may increase bile viscosity and obstruct bile flow, creating a microenvironment conducive to persistent bacterial colonization and biofilm maturation. In contrast, physiologically, the biliary biofilm, if present, is sparse, loosely adherent, and characterized by low-viscosity EPS with minimal polysaccharide content. Such biofilms exert negligible effects on bile dynamics or immune clearance, underscoring the importance of EPS composition and density in determining its pathological potential (Adcox et al., 2016; Prouty et al., 2002).

At the molecular level, proteomic studies have further demonstrated that P. aeruginosa biofilms in bile are enriched with outer membrane proteins such as OmpA and chaperone proteins such as DnaK, which enhance adhesion and bile tolerance, providing novel insights into the persistence of infection in the biliary tract (Yang et al., 2023). Xiao et al. (2024) found the enrichment of six pathways in CBD stones among ten significantly different Kyoto Encyclopedia of Genes and Genomes pathways, especially the biofilm formation pathway associated with *P. aeruginosa*. Beyond clinical associations, mechanistic insights suggest that modulation of the purine–c-di-GMP signaling system represents a potential strategy to inhibit P. aeruginosa biofilm maturation, thereby attenuating its lithogenic potential (Kennelly et al., 2024).

As summarized in Table 4, biofilms and their extracellular matrix play multifaceted roles in GSD pathogenesis, manifesting in the initiation of stone nucleation, and sustenance of bacterial survival within an inflamed biliary environment. Importantly, it is not merely the presence of biofilms but the pathological remodeling of their EPS components, particularly under dysbiotic or inflammatory stimuli, ultimately driving the transition from colonization to lithogenesis. The accumulation of charged polysaccharides, proteins, and DNA within the biofilm matrix may enhance bacterial adhesion, resist bile salt-mediated degradation, and enhance the aggregation and retention of lithogenic substrates (Costerton et al., 1999; Donlan and Costerton, 2002; Flemming et al., 2007; Karygianni et al., 2020; Nicoletti et al., 2020; Terada et al., 2012; Wang et al., 2018). These structural and biochemical features of EPS-enriched biofilms may contribute to the formation of GSD, persistence, and recurrence, thus implicating biofilm modulation as a potential target for therapeutic intervention. In addition to *P. aeruginosa*, bacterial genera such as *E. coli*, *Klebsiella pneumoniae, Enterococcus, C. perfringens, Bacteroides*, and *Acinetobacter* are also contributors of biofilm formation in the biliary tract, exhibiting intimate associations with GSD development (Grigor’eva and Romanova, 2020).

**TABLE 4:** The role of biofilms and their extracellular matrix in the pathogenesis of GSD.

Literature	Functionality	Relevance to biliary biofilms	EPS components involved
Wang et al., 2018	Adhesion	Promote the colonization of pathogenic bacteria (e.g., *E. coli*, and *Enterococci*) on the surface of gallbladder mucosa, cholesterol crystals, etc., and establish the initial structure of biofilm.	Polysaccharides, proteins, DNA
Karygianni et al., 2020	Bacterial cell aggregation	Promote the aggregation of bacterial communities to form three-dimensional structures, enhance biofilm stability and drug resistance.	Polysaccharides, proteins
Wang et al., 2018	Polyframe components of biofilms	Provide biofilm scaffolding structure, determine biofilm thickness and densification, and promote stone core formation.	Neutral and charged polysaccharides, proteins, and DNA
Costerton et al., 1999	Protective barrier	Resistant to destruction by bile components (e.g., bile salts), immune factors, and antibiotics, and maintain bacterial survival within the biofilm.	Polysaccharides and proteins
Flemming et al., 2007	Adsorption of organic compounds	Absorb bilirubin, cholesterol, Ca^2+^ and other components to provide substrate for deposition of gallstone components.	Charged or polar polysaccharides and proteins
Terada et al., 2012	Depositional attachment of inorganic material	Provide negatively charged structures to bind calcium salts and promotes deposition of inorganic crystals such as calcium bilirubinate.	Charged polysaccharides or proteins that bind calcium and iron salts
Nicoletti et al., 2020	Viscosity	Increase the local viscosity of bile, slow down the flow of bile, thus contributing to the deposition and aggregation of stone material.	Mucopolysaccharides (e.g., acidic mucopolysaccharides)
Donlan and Costerton, 2002	Pooling of diversity	Aggregate a variety of microorganisms and their metabolites, jointly participating in the process of GSD formation.	Polysaccharides, and glycoproteins

Similar to *E. coli*, *P. aeruginosa* can harbor multiple efflux pump systems on its surface, among which the MexAB-OprM pump from the RND family plays a particularly important role (Ahmadian et al., 2023). This bacterium can also produce b-G, the mechanisms of which are detailed in the previous text. In addition, *P. aeruginosa* may also shorten the time required for cholesterol crystallization in bile models, although the exact mechanism remains poorly understood (Zhu et al., 2009).

***Desulfovibrionales***. In the context of cholesterol GSD, Hu et al. demonstrated that gut microbiota, particularly bacteria of the *Desulfovibrionales* order, play a critical role in gallstone formation (Hu et al., 2022). The authors reported that *Desulfovibrionales* were enriched in the feces of patients with cholesterol GSD as well as in gallstone-susceptible mice (Hu et al., 2022). Notably, fecal microbiota transplantation from these patients to gallstone-resistant mice resulted in gallstone formation, indicating a causal relationship (Hu et al., 2022). Mechanistically, this effect was associated with increased production of secondary bile acids in the cecum, which increased bile hydrophobicity and enhanced intestinal cholesterol absorption. Furthermore, H2S produced by *Desulfovibrionales* was shown to activate hepatic FXR, mechanistically associated with suppression of cholesterol 7α-hydroxylase (CYP7A1) expression and bile acid synthesis (Huang et al., 2024; Ye et al., 2022). As a consequence, hepatic cholesterol transporters Abcg5 and Abcg8 were upregulated, promoting biliary cholesterol secretion and facilitating gallstone formation (Huang et al., 2024; Ye et al., 2022). These findings underscore the significant role of specific gut bacterial taxa in modulating bile acid metabolism and biliary cholesterol homeostasis, thereby contributing to GSD pathogenesis.

### Firmicutes

***Enterococcus.*** *Enterococcus* in the biliary tract has been unveiled to be closely associated with the formation of GSD, as has been identified by Xiao et al. (2024) via 16S rRNA gene sequencing. Similarly, *Enterococcus* has likewise been detected in the bile of GSD patients (Han et al., 2021; Kim et al., 2021; Saab et al., 2021; Shen et al., 2015; Xiao et al., 2021). *Enterococcus* is an opportunistic pathogen that is typically absent in the healthy biliary tract and primarily resides in the intestine, where it contributes to digestion and vitamin synthesis (Krawczyk et al., 2021). Intestinal *Enterococcus* may retrogradely translocate into the bile ducts and proliferate in cases of biliary obstruction, impaired bile flow, or immunosuppression, which is consistent with the duodeno-biliary reflux hypothesis proposed by several researchers (Han et al., 2021; Kim et al., 2021; Lyu et al., 2021). *Enterococcus* is frequently implicated in acute cholangitis and malignant biliary obstruction (Karasawa et al., 2021). It can tolerate a wide range of pH values (4.5–10.0) and high sodium chloride concentrations, giving it strong adaptive capacity and pathogenic potential in new environments (Karasawa et al., 2021). *Enterococcus* can express quorum-sensing-regulated surface proteins that mediate adhesion to the extracellular matrix and confer resistance to phagocytosis, enhancing its persistence and pathogenicity within the biliary tract (Coburn and Gilmore, 2003; Ike and Clewell, 1992). Microbial surface components recognizing adhesive matrix molecules on *Enterococcus* can bind to host collagen, facilitating the colonization of biological surfaces and evading immune detection (Kao and Kline, 2019). *Enterococcus* can also secrete BSH (Franz et al., 2001), which disrupts bile acid homeostasis and promotes inflammation, contributing to the formation of GSD. Other bacteria such as *Listeria* (Dussurget et al., 2002), *Clostridium* (Gopal-Srivastava and Hylemon, 1988), *Bacteroides* (Kawamoto et al., 1989), and *Bifidobacterium* (Grill et al., 1995) can also produce BSH and participate in lithogenesis. In addition, *Enterococcus* in the biliary tract may also exert a GSD-promoting role through the production of β-G that facilitates brown pigment stones formation, and biofilm formation as well (Krawczyk et al., 2021; Shankar et al., 1999).

*Clostridium perfringens* **(*C. perfringens*).** *C. perfringens* in bile exhibits PLC activity, which can hydrolyze PC into fatty acids, indirectly contributing to the formation of GSD (Cetta, 1986; Nakano et al., 1988). Moreover, there are significant interspecies differences in enzyme activity, although many bacterial species are capable of producing β-G. Notably, the β-G activity of *C. perfringens* is reported to be 34 times higher than that of *E. coli* (Leung et al., 2001). In addition, *C. perfringens* may also participate in the development of GSD through the production of cell-associated mucus that contributes to biofilm formation.

### Bacteroidetes

***Bacteroides.*** GSD patients have been found to exhibit a significantly elevated relative abundance of *Bacteroidetes* in their bile or intestinal microbiota. The metabolic activities of this phylum are closely associated with bile component imbalance, local inflammation, and eventually the formation of GSD (He et al., 2025). *Bacteroides* can produce β-G to stimulate the formation of pigment stones (Ye et al., 2016). With BSH activity, it can also induce cholesterol supersaturation in bile and facilitate cholesterol crystal formation (Kawamoto et al., 1989). *Bacteroides* may also induce the formation of biofilms in the biliary tract (Grigor’eva and Romanova, 2020), providing a structural scaffold for stone development. Its lipopolysaccharides (LPS) can induce mucosal inflammation and stimulate biliary epithelial cells to secrete large amounts of mucus, thereby accelerating the nucleation and growth of GSD.

### Actinobacteria

***Actinomyces.*** Chronic inflammation is a well-established contributor to the development of bile duct stones (Lyons et al., 2010). By inducing regulatory T cells, *Actinomyces* has been suggested to act as a potential regulator of inflammation and immune responses. Xiao et al. (2024) documented a markedly reduced abundance of *Actinobacteria* in patients with CBD stones, suggesting a weakened biliary immune barrier and impaired inflammatory regulation, potentially creating a microenvironment favorable to stone formation. In addition, *Actinomyces* can ferment carbohydrates to produce short-chain fatty acids (SCFAs) (e.g., acetate, propionate, and butyrate). These SCFAs serve as important energy sources for epithelial cell renewal, with butyrate standing out for its critical role in defending against bacterial toxins (Song et al., 2006). As previously discussed, the depletion of these “beneficial bacteria” may facilitate the progression of GSD.

## The relationship between gut microbiota and GSD

The GI tract, harboring both commensal and pathogenic bacteria (Binda et al., 2022), is regarded as one of the largest microbial reservoirs in humans. From an embryological perspective, the biliary system and the duodenum both originate from the primitive foregut, and the anatomical connection between the CBD and duodenum (i.e., the bile drainage pathway) is established early in development, highlighting their close relationship in both structure and function (Yeo et al., 2023). Therefore, in order to elucidate their potential roles in the pathogenesis of GSD, it is rational to investigate the relationship between the biliary microbiota and the duodenal—and even the entire intestinal—microbiome. Some researchers argue that the sphincter of Oddi, as a physiological barrier, can minimize the influence of gut microbiota on the biliary tract. However, human microbial ecosystems actually constitute a dynamic, interconnected network of microbial communities, instead of existing in isolation (Costello et al., 2009). Therefore, the biliary and intestinal microbiota, rather than being accepted as entirely separate systems, should be considered to be intrinsically linked. Numerous diseases, including Crohn’s disease, colorectal cancer, and metabolic disorders, have established a strong association with gut microbiota dysbiosis (Kanehisa, 2019). Wu et al. (2013) analyzed microbial communities in the intestines, bile, and gallstones of 29 GSD patients, along with fecal microbiota from 38 healthy controls. For the first time, these authors reported a dysbiotic gut microbiota profile in GSD patients, supporting a contributing role of microbiome imbalance in the GI tract, particularly in cholesterol gallstone formation (Wu et al., 2013). With the establishment of a murine model of cholesterol gallstones, Wang et al. (2017) observed reduced gut microbial richness and diversity in animals fed by a lithogenic diet. Specifically, there was a notable decline in the abundance of *Firmicutes* and a significant reduction in the *Firmicutes*-to-*Bacteroidetes* ratio; moreover, compositional differences were observed at the phylum, family, and genus levels, suggesting a strong link between gut dysbiosis and cholesterol gallstone pathogenesis in mice (Wang et al., 2017). Both Lyu et al. (2021) and Han et al. (2021) reported high similarity between the biliary microbiota of patients with CBD stones and that of duodenal fluid, with all detected biliary bacteria also found in the upper GI tract. These findings support the duodeno-biliary reflux theory as a significant mechanism in the formation of GSD (Han et al., 2021; Lyu et al., 2021). It is hypothesized that the sphincter of Oddi functions as an anatomical barrier, which can normally prevent retrograde bacterial migration from the intestine into the bile ducts. This barrier may become compromised in patients with biliary disease, allowing intestinal bacteria to reflux into the biliary tract, alter the local microenvironment, and eventually promote the formation of GSD. This concept was further validated by Kamada et al. (2013). Together, the gut microbiota can trigger the development of GSD through several key mechanisms, as outlined below.

(1) Influence on enterohepatic circulation and bile acid metabolism

**FIGURE 2:**
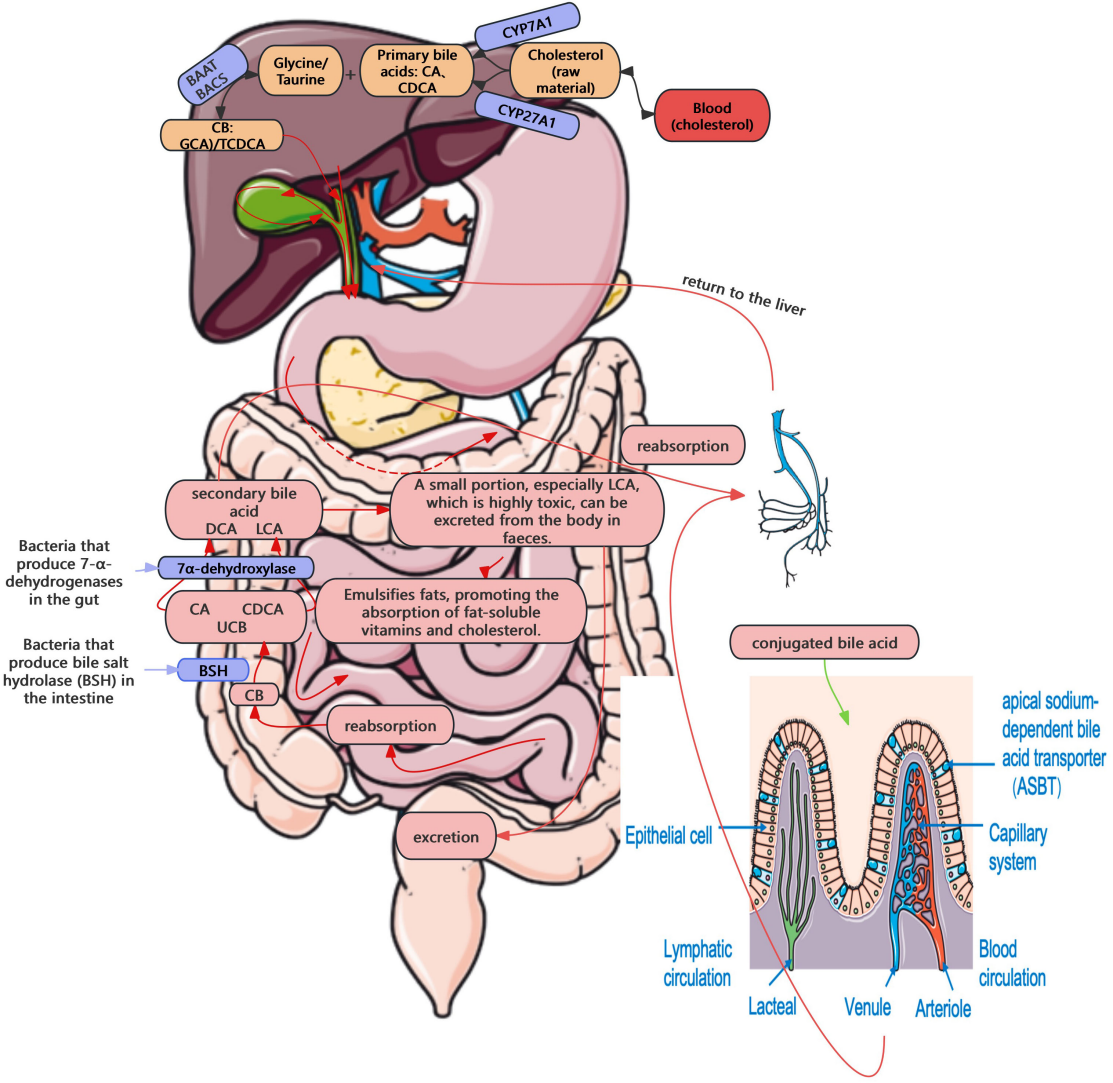
Enterohepatic circulation.

The enterohepatic circulation of bile acids plays a central role in cholesterol homeostasis and GSD pathogenesis (Cai and Chen, 2014). In this process, cholesterol is converted into primary bile acids, e.g., and chenodeoxycholic acid (CDCA), conjugated, secreted into bile, and recycled via intestinal reabsorption and hepatic uptake—functions tightly regulated by enzymes, including CYP7A1 and sterol 27-hydroxylase (CYP27A1), and transporters, including bile salt export pump (BSEP), apical sodium-dependent bile acid transporter (ASBT), and sodium-taurocholate cotransporting polypeptide (NTCP) (Cai and Chen, 2014). A small portion of bile acids that are not reabsorbed reach the colon, where they are deconjugated by gut bacteria expressing BSH, producing free bile acids (Wise and Cummings, 2022). Subsequently, they are converted by 7α-dehydroxylase into secondary bile acids, such as deoxycholic acid (DCA) and lithocholic acid (LCA) (Wise and Cummings, 2022). While some of these are reabsorbed into the portal system, others, particularly the more toxic LCA, are excreted in feces (Wise and Cummings, 2022). This enterohepatic circulation occurs 6–10 times per day, with a recycling efficiency of over 95% (Roberts et al., 2002; Figure 2). Enterohepatic circulation becomes disrupted in the presence of gut microbiota dysbiosis. A recent study also demonstrated that intestinal flora imbalance significantly affects bile acid metabolism, contributing to gallstone formation (Wang et al., 2020; Zhao et al., 2023). Elevated BSH activity may interfere with bile acid reabsorption, resulting in decreased bile salt concentration, reduced cholesterol solubility, and ultimately, the precipitation of cholesterol crystals, leading to the formation of GSD.

The farnesoid X receptor (FXR)–fibroblast growth factor 15 (FGF15) signaling axis is also pivotal in gut microbiota–mediated GSD pathogenesis, functioning as a classic negative feedback loop. Within the enterohepatic cycle, CYP7A1 is a rate-limiting enzyme in bile acid synthesis. In the intestine, bile acids bind and activate the FXR, further inducing the expression of small heterodimer partner (SHP) and fibroblast growth factor 15/19 (FGF15/19), in turn inhibiting intrahepatic CYP7A1 expression, thereby reducing bile acid synthesis (Song et al., 2022). During biliary microbiota dysbiosis, increased BSH activity has been shown to elevate the level of free bile acids in the intestine, further triggering the FXR–FGF15 feedback loop, and suppressing bile acid synthesis. The resultant cholesterol supersaturation, in conjunction with persistent dysbiosis, might create a vicious cycle that significantly increases the risk of cholesterol gallstone pathogenesis.

In addition, bacterially derived secondary bile acids (e.g., DCA and LCA) may activate the G protein-coupled bile acid receptor Takeda G-protein-coupled receptor 5 (TGR5), and increase the intracellular cAMP levels, eventually enhancing gallbladder relaxation and delaying bile emptying. Prolonged bile stasis may facilitate cholesterol precipitation, and consequently increase the risk of cholesterol gallstone formation. Simultaneously, TGR5 can modulate both gallbladder motility and local inflammatory responses, thus influencing stone formation (Keitel et al., 2009). TGR5 can also synergize with FXR to regulate the cholesterol efflux pump BSEP. Dysfunction in this pathway may impair cholesterol secretion, result in cholesterol supersaturation in bile, and accelerate stone formation (Li and Chiang, 2014). In addition, TGR5 can regulate anti-inflammatory effects; while its impairment may reduce these defenses, allowing inflammation to alter bile composition and promote cholesterol crystallization (Ye et al., 2024).

(2) Effects of microbial metabolites on bile composition and cholesterol saturation

Trimethylamine N-oxide (TMAO) is a microbial metabolite derived from dietary choline, L-carnitine, and PC, which are converted into trimethylamine by gut microbes and subsequently oxidized to TMAO in the liver by flavin-containing monooxygenase 3 (Janeiro et al., 2018). Existing clinical data reveals significantly elevated serum TMAO levels in individuals with GSD (Chen et al., 2019). TMAO enables the activation of the FXR–FGF15/SHP–FGFR4 signaling axis, thereby suppressing bile acid synthesis and promoting cholesterol supersaturation in bile (Ding et al., 2018). In addition, fermentation of carbohydrates by the gut microbiota can produce SCFAs such as acetate, propionate, and butyrate. Conversely, gut microbiota dysbiosis reduces SCFA production, thereby increasing the risk of GSD.

(3) Duodeno-biliary reflux theory

Intestinal bacteria may retrogradely translocate into the biliary tract via the sphincter of Oddi, thereby contributing to the formation of GSD. This mechanism has been discussed in detail in the previous text.

(4) Regulation of lipid and host energy metabolism

The gut microbiota can also modulate hepatic expression of lipid metabolism–related genes, such as SREBP-1c and LXRα, thereby regulating cholesterol synthesis and excretion (Wang W. et al., 2025), and ultimately promoting cholesterol gallstone pathogenesis.

## Other bacterial mechanisms in the formation of GSD

In addition to gut and biliary microbiota, oral and parasitic-associated microbiota as well as other bacterial sources have also been implicated in GSD pathogenesis, suggesting a broader microbial network beyond the hepatobiliary axis. Oral bacteria may interfere with the biliary environment either directly via hematogenous spread, or indirectly by altering gut microbiota composition (Velsko et al., 2014). For example, *Synergistetes*, a phylum commonly associated with periodontal disease, was detected in high abundance in the bile of GSD patients, but was absent in individuals without hepatobiliary disease, as reported by Lyu et al. (2021). These bacteria are known to produce proteolytic enzymes that can degrade epithelial junctions and induce mucin overproduction, both of which may promote crystal aggregation and bile stasis, two hallmark features of GSD formation (Saltykova et al., 2016). Additionally, bacteria symbiotically associated with parasitic organisms can modulate host bile acid profiles or trigger localized immune responses in the biliary tract, thereby disturbing the physicochemical stability of bile and favoring lithogenesis (Yu et al., 2016). These alternative microbial pathways underscore the multifactorial and trans-compartmental nature of GSD.

Moreover, LPS, an essential structural component of the outer membrane of Gram-negative bacteria, has recently been implicated in GSD pathogenesis. Through activation of the TLR4/NF-κB signaling cascade, LPS induces local inflammation and mucin hypersecretion, thereby facilitating cholesterol crystal nucleation and biliary stasis (Yu et al., 2024b). These findings highlight LPS as an additional microbial factor linking Gram-negative bacterial infection to gallstone formation (Yu et al., 2024b).

## Conclusion and future perspectives

Gallstone disease is currently viewed as the outcome of multifactorial interactions between host factors and complex microbial ecosystems, rather than a mere physicochemical imbalance in bile. This review highlights the biliary and intestinal microbiota as interconnected components of a broader biliary–intestinal microbial axis, whose dysregulation synergistically drives lithogenesis. In the biliary tract, specific phyla such as *Proteobacteria* (e.g., *E. coli*, *P. aeruginosa*) and *Firmicutes* (e.g., *Enterococcus*, *C. perfringens*) can promote stone formation via β-G secretion, phospholipid hydrolysis, efflux pump activation, and biofilm development. These mechanisms may disrupt bile composition, impair flow, and trigger inflammation. *Bacteroidetes* (e.g., *Bacteroides*) further participate in this process by producing BSH, LPS, and PLs that destabilize bile acid homeostasis and induce mucin secretion. Conversely, *Actinobacteria* (e.g., *Actinomyces*), through PLs production and barrier maintenance, may exert protective roles; while their depletion may favor stone formation. Meanwhile, the gut microbiota can also regulate bile composition and gallbladder motility via the enterohepatic circulation and signaling pathways such as FXR–FGF15 and TGR5. Intestinal dysbiosis can reduce bile acid recycling, increase cholesterol supersaturation, and impair gallbladder emptying—systemic disturbances that complement biliary microbial shifts. Therefore, GSD pathogenesis should be interpreted preferably as the result of dual-site dysbiosis.

This integrated view in our study holds clinical promise. Specifically, microbial profiling of bile and feces may aid risk stratification, while identifying specific bacterial signatures (e.g., β-G producers, and biofilm-forming taxa) can help predict recurrence or complications. Targeted microbial interventions (e.g., probiotics, bacteriophage therapy, fecal microbiota transplantation, etc.) may offer adjunctive strategies. Nevertheless, these applications remain exploratory, limited by methodological variability and a lack of clinical validation.

With regard to microbial interventions, although clinical evidence in GSD remains limited, it is nonetheless informative. A small randomized pilot trial evaluated probiotic supplementation, showing modest improvements in bile acid composition but no significant effect on gallstone recurrence (Prete et al., 2020; Sivamaruthi et al., 2020). Isolated observational reports have described altered biliary microbiota following fecal microbiota transplantation (FMT) in patients with recurrent biliary tract infections, although direct benefits for GSD prevention or treatment remain unproven (Hu et al., 2022). Moreover, to date, no controlled clinical trial has assessed phage therapy in GSD, leaving its application largely theoretical. Collectively, these small-sample trials and observational studies highlight both the promise and limitations of microbial interventions, thereby underscoring the need for larger, well-designed clinical studies in GSD populations.

Looking forward, future research should prioritize longitudinal, multi-center studies that integrate multiple methods, including multi-omics, spatial microbiota mapping, and metabolite profiling. Microbial contributions can be further clarified by employing gnotobiotic models and bile duct-mimicking organoids. Ultimately, such approaches may enable a deeper understanding of the biliary–intestinal microbiota axis and facilitate the development of precise, microbiota-informed strategies to prevent and treat GSD clinically.
